# Correction: SARS-CoV-2 transmission routes from genetic data: A Danish case study

**DOI:** 10.1371/journal.pone.0261892

**Published:** 2021-12-21

**Authors:** The PLOS ONE Staff

## Notice of republication

This article was republished on November 4, 2020, to correct an error in the title: “SARS-CoV-2 transmission routes from genetic data: A Danish case study” was erroneously displayed as “SARS-CoV-2 transmission routes from genetic data: A Danishcase study”. The publisher apologizes for the error. Please download this article again to view the correct version. The originally published, uncorrected article and the republished, corrected article are provided here for reference.

## Supporting information

S1 File Originally published, uncorrected article. (PDF)

S2 File Republished, corrected article. (PDF)
