# Supplementary material for: SARS-CoV-2 transmission routes from genetic data: A Danish case study
Source: PLoS One. 2020 Oct 29;15(10):e0241405. doi: 10.1371/journal.pone.0241405 (PMC7595399; doi:10.1371/journal.pone.0241405)
Supplement: S1 File — (PDF) [file pone.0241405.s001.pdf]

SARS-COV-2 TRANSMISSION ROUTES FROM GENETIC DATA: A DANISH CASE STUDY SUPPLEMENTARY MATERIAL

ANDREAS BLUHM, MATTHIAS CHRISTANDL, FULVIO GESMUNDO, FREDERIK RAVN KLAUSEN, LAURA MANČINSKA, VINCENT STEFFAN, DANIEL STILCK FRANÇA, AND ALBERT H. WERNER

S1 Discussion on additional common Danish mutations

In this section, we discuss some of the most common Danish mutations (with respect to the Wuhan reference) in addition to the ones already discussed in the main text. We will use another haplotype from [27], namely A8. This haplotype corresponds to G1440A and G2891A with respect to the Wuhan reference sequence. We refer to Tables S1 and S2 for a summary of mutations occurring at least three times in the Danish data.

S1.1 Mutation C7011T. Worldwide there are 64 sequences with the mutation C7011T, of which 38 exhibit haplotype A8. Among these 38 are 13 Danish sequences.

From the following analysis, we suspect that the mutation occurred independently at least twice. In addition to the 38 sequences, there are 18 sequences, all from the UK, that have the mutation C7011T co-occurrent with the haplotype A1a1b, but not with A8. In the worldwide data set (as of May 26) the haplotype A1a1b appears 645 times and A8 appears 310 times. Thus, if the mutation C7011T had not happened twice independently, it must have happened before the mutations corresponding to A1a1b and A8. However, this would not account for the much larger number of sequences with A1a1b and A8 not having the mutation C7011T in addition. To explain this, both A1a1b (comprising 4 mutations) and A8 (comprising 2 mutations) must have occurred independently at least twice (in sequences with both C and T at the site 7011). This is however less plausible than a single nucleotide mutation occurring several times. Therefore we conclude that C7011T happened independently twice.

One occurrence seems to be from the UK; the other cluster (the one co-occurrent with A8) is less clear. The cluster with the Danish sequences contains five Austrian sequences. The Austrian sequences are from Innsbruck, Tyrol, Ischgl, Vienna and St. Anton. This means that four out of five are from the region of Tyrol, but they are not concentrated in Ischgl. In the overview of the Austrian haplotypes in Fig S2 the haplotype A8 is missing in three of the Austrian sequences, but they all have at least one of the two mutations constituting A8. Some of the sequences have an ambiguity character here, so it could be that most Austrian sequences have A8.

S1.2 Mutations T8788C and G26951A. Worldwide there are 55 sequences with the mutation T8788C and 56 with mutation G26951A. Almost all are Danish.

In Fig S1 one can also see that those two mutations are co-occurring and that they are in the beginning of several transmission chains of mutations.

S1.3 Mutation C7834T. Worldwide there are 73 sequences with the mutation C7834T, of which 66 are with haplotype A2a. Among those are the 60 Danish ones so the mutation C7834T constitutes one most abundant Danish mutations and in Fig S1 one can see that they are starting chains of mutations.

S1.4 Mutation G24368T. Worldwide there are 163 sequences with the mutation G24368T, of which 156 are with haplotype A2a2a. Among those are the five Danish ones (all with haplotype A2a2a). The majority of the others are from the UK (89), Sweden (50), the Netherlands (4), Iceland (3). The three Icelandic samples all exhibit a travel history connected to the UK. The mutation is non-synonymous and is located in the spike protein. The mutation G24368T seems closely tied to the UK and we note that compared to the total number of sequences, a much larger fraction of the Swedish sequences is of this type (50/163) compared to the corresponding fraction of Danish sequences (5/582). This indicates that Sweden might have been infected through the UK to a larger extend than Denmark. Another point of interest is that G24368T is co-occurring with haplotype A2a2a. This is a specific example that the link between haplotype A2a2a and Austria is non-exclusive.

S2 Supplementary tables

Total	World 20239	Denmark 582	Sweden 163	Norway 33	France 375	Italy 84	UK 6668	Netherlands 489	Austria 247	Germany 173
A	20220	580	162	32	374	83	6645	488	246	172
A1a	1418	0	1	6	11	7	901	50	4	6
A1a1	1372	0	1	1	9	0	898	50	4	6
A1a1a	546	0	0	0	2	0	271	45	3	1
A1a1a1	42	0	0	0	0	0	0	0	0	0
A1a1a2	14	0	0	0	0	0	0	0	0	0
A1a1a3	8	0	0	0	0	0	0	0	0	0
A1a1a4	84	0	0	0	0	0	59	0	0	0
A1a1a5	27	0	0	0	1	0	15	0	0	0
A1a1b	645	0	1	0	7	0	523	2	0	5
A1a2	0	0	0	0	0	0	0	0	0	0
A2	13900	553	153	25	332	76	5024	314	211	141
A2a	13867	552	153	25	332	76	5024	314	211	126
A2a1	4363	38	43	3	26	36	2690	110	96	50
A2a1a	363	13	1	0	2	1	85	83	34	17
A2a1a1	27	0	0	0	0	0	0	0	0	0
A2a1a2	28	0	0	0	0	0	7	4	0	8
A2a1a3	12	1	0	0	0	0	8	0	0	0
A2a1b	349	4	1	0	0	0	280	2	2	2
A2a1c	230	0	1	0	1	0	153	5	4	0
A2a1d	6	0	0	0	0	0	0	0	0	0
A2a2	5343	410	70	14	185	0	421	52	66	44
A2a2a	4343	405	66	12	112	0	279	34	63	44
A2a3	943	13	3	1	1	2	425	19	22	2
A2a3a	82	0	0	0	0	0	0	0	0	0
A2a4	13	0	0	0	0	0	0	0	0	0
A2a5	195	3	3	6	1	5	63	5	0	0
A2a6	104	4	0	0	0	0	22	48	5	2
A2a7	432	0	0	0	0	0	409	0	0	0
A2a8	513	1	0	0	110	0	43	6	1	2
A2a9	136	1	1	0	2	6	51	7	2	6
A2a10	125	0	0	0	0	0	92	0	0	0
A2a11	23	0	0	0	0	0	2	19	0	0
A2a12	26	0	0	0	0	1	7	5	0	0
A2a13	149	2	0	0	0	0	131	0	0	0
A3	162	1	1	1	0	0	2	2	0	1
A6	158	2	0	0	0	0	19	99	3	1
A7	17	0	0	0	0	0	4	0	0	0
A8	310	16	3	0	0	0	189	6	5	17
A9	73	0	0	0	0	0	63	0	0	0
A10	3	0	0	0	0	0	0	0	0	0
B	2173	1	2	0	2	0	72	7	5	2
B1	1299	0	0	0	0	0	5	0	0	0
B1a	1275	0	0	0	0	0	5	0	0	0
B1a1	1249	0	0	0	0	0	5	0	0	0
B1a1a	124	0	0	0	0	0	0	0	0	0
B1a1a1	13	0	0	0	0	0	0	0	0	0
B1a1a1a	10	0	0	0	0	0	0	0	0	0
B1a1a1b	1	0	0	0	0	0	0	0	0	0
B2	46	0	0	0	0	0	0	0	0	1
B4	98	0	2	0	0	0	0	3	0	0

TABLE S1. **Summary of haplotypes for selected European countries as per May 26, 2020.** We include the haplotypes described in [27].

	World	Denmark	Sweden	Norway	France	Italy	UK	Netherlands	Austria	Germany
Total	20239	582	163	33	375	84	6668	489	247	173
C1302T	115	103	6	0	0	0	0	0	0	0
C7834T	73	60	0	0	0	0	4	0	0	0
G26951A	56	54	0	0	0	0	2	0	0	0
T8788C	55	53	0	0	0	0	2	0	0	0
C15842A	20	20	0	0	0	0	0	0	0	0
C12781T	37	17	0	0	0	0	0	1	0	0
G15380T	40	16	0	1	1	0	5	0	8	2
C7011T	64	13	2	0	0	0	28	1	5	0
C21707T	51	11	0	0	0	0	4	0	0	0
C11074T	48	11	0	0	0	0	19	1	0	0
C29200T	41	8	0	0	0	0	21	0	0	0
G22103C	8	8	0	0	0	0	0	0	0	0
C28253A	8	7	0	0	0	0	0	0	0	0
A6825C	10	6	0	1	0	0	1	1	1	0
C3743T	8	6	0	0	0	0	1	0	0	0
C16887T	72	5	1	0	3	0	32	0	0	0
G19086T	13	6	0	0	0	0	3	0	0	1
G24368T	163	5	50	0	1	0	95	4	0	0
T99C	4	4	0	0	0	0	0	0	0	0
C218T	8	4	0	0	0	0	1	0	0	0
A518G	7	3	0	0	0	0	0	0	0	0
T658C	26	4	0	0	0	0	1	4	0	2
C4002T	174	4	1	0	0	0	118	2	2	0
A4548G	3	3	0	0	0	0	0	0	0	0
C4668T	23	3	0	0	0	0	16	1	0	0
C6539T	8	6	0	0	1	0	1	0	0	0
C7029T	4	3	0	0	0	0	0	0	0	0
C7819T	10	3	0	0	1	0	0	0	0	0
A9280G	8	3	0	0	0	0	0	0	0	0
C9803T	16	3	0	0	1	0	1	1	0	0
C9979T	15	4	0	0	0	0	7	0	0	0
G10465A	11	5	0	0	0	0	0	0	0	0
C13536T	190	4	1	0	0	0	118	2	2	2
G15906A	11	4	0	0	0	0	0	0	0	0
C18744T	29	5	0	0	0	0	3	0	0	0
C18877T	595	3	1	2	0	0	30	9	7	0
C19263T	15	3	0	0	0	0	1	0	0	0
G20398A	7	3	0	0	0	0	0	0	0	0
G20419T	12	4	3	1	0	0	0	0	1	0
G20887A	10	4	0	0	2	0	0	0	0	1
C21742T	21	5	0	0	0	0	2	0	0	0
C21855T	18	9	0	0	0	0	1	0	0	0
T21976C	8	4	0	0	0	0	0	0	0	0
A23975G	4	4	0	0	0	0	0	0	0	0
G25785T	15	3	1	0	0	0	3	0	0	0
C28629T	20	4	0	0	0	0	1	4	1	2
G28899T	8	4	0	0	0	0	2	0	0	0
C29719T	6	3	0	0	0	0	2	1	0	0
G29734C	171	3	1	0	0	0	64	9	9	1
G29779T	13	4	0	0	0	0	5	0	0	0
C23575T	29	3	1	1	1	1	9	0	0	0
C23587T	17	3	0	1	0	0	6	0	0	0
ATGA1605A	484	9	1	0	2	0	306	108	0	1
C29095T	71	7	0	0	0	0	13	0	0	1
C7164T	12	2	0	0	0	0	5	1	0	0
C619T	21	2	0	0	0	0	0	0	0	0
C25499T	2	2	0	0	0	0	0	0	0	0

TABLE S2. **Summary of mutations for selected European countries as per May 26, 2020.** We include all mutations with respect to the Wuhan reference that appears at least three times in the Danish data. The last three mutations are included because they are relevant for the discussion in the section on Chains of mutations in Denmark.

S3 Supplementary figures

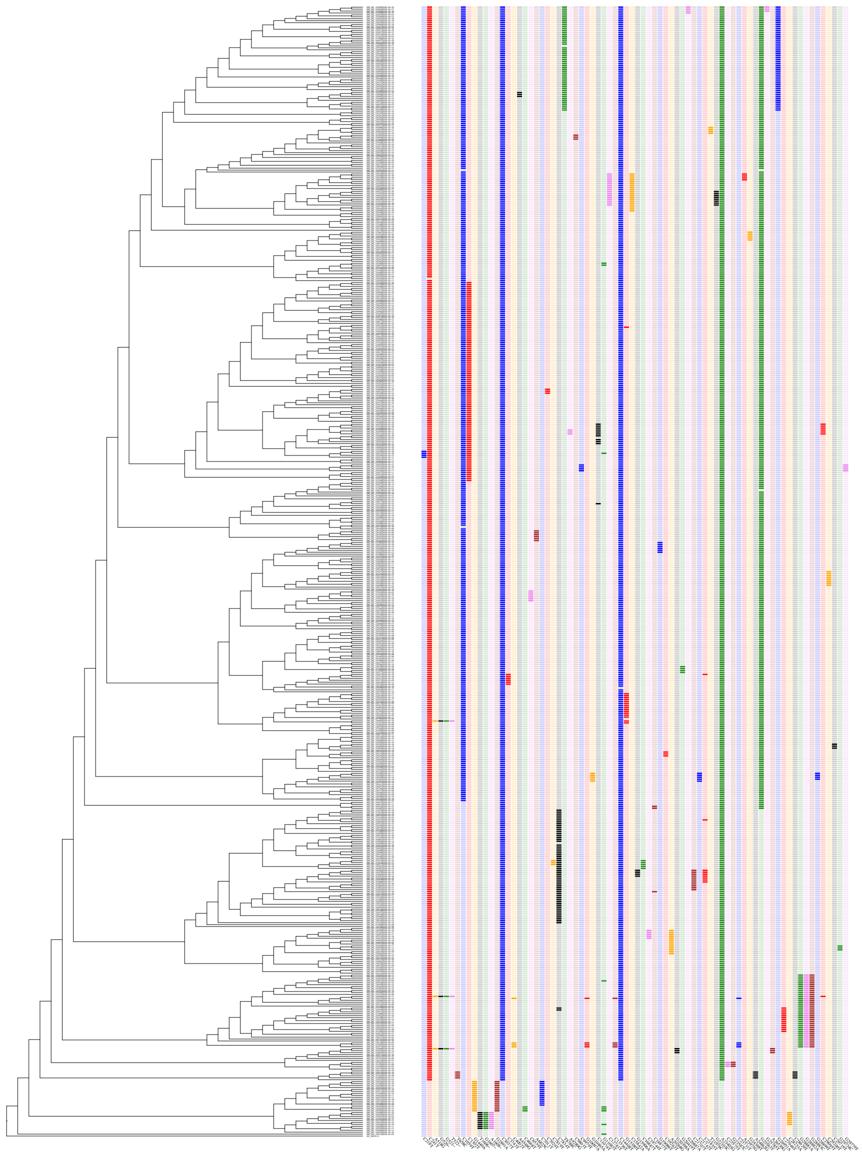

Fig S1. **Phylogenetic tree with Danish sequences.** Overview of the phylogenetic tree of Denmark on the left with the corresponding mutations on the right. Displayed are only mutations which happen at least thrice. The plot shows how about 70% of the sequences have the haplotype A2a2a (corresponding to mutations C241T, C3037T, A23403G, C14408T, G25563T, C1059T) and that 103 have the further mutation C1302T. Note that the mutation A518G with the co-occurring deletion 520-522 is equivalent to the deletion 518-520 which is listed in CoV-GLUE [35] as can easily be seen by inspecting the Wuhan sequence.

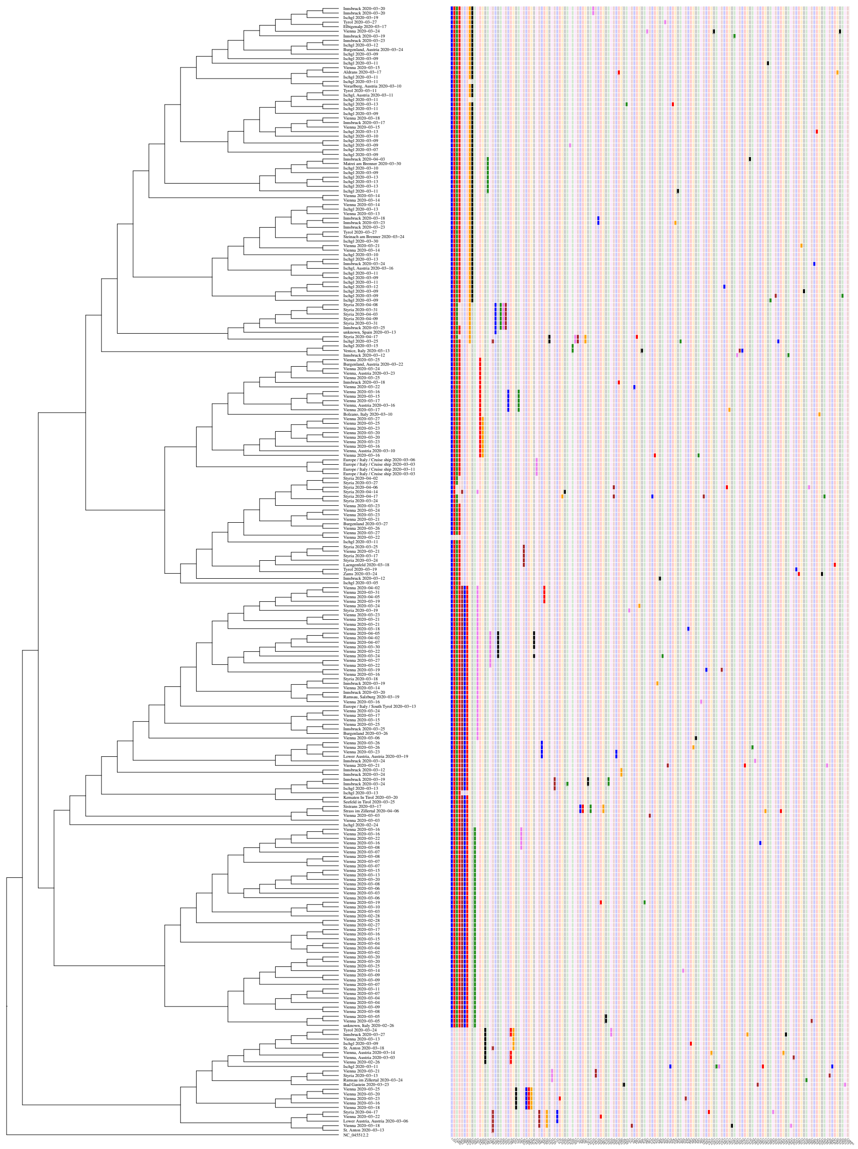

FIG S2. **Phylogenetic tree with Austrian sequences.** Austrian sequences with location metadata and mutations with respect to the Wuhan standard reference. Notice the match between the sequences with haplotype A2a2a and the Ischgl location metadata. Note also the match between the haplotype A8 (G1440A and G2891A) as well as the mutation C7011T and the region of Tyrol. Both mutations are also present in Denmark.

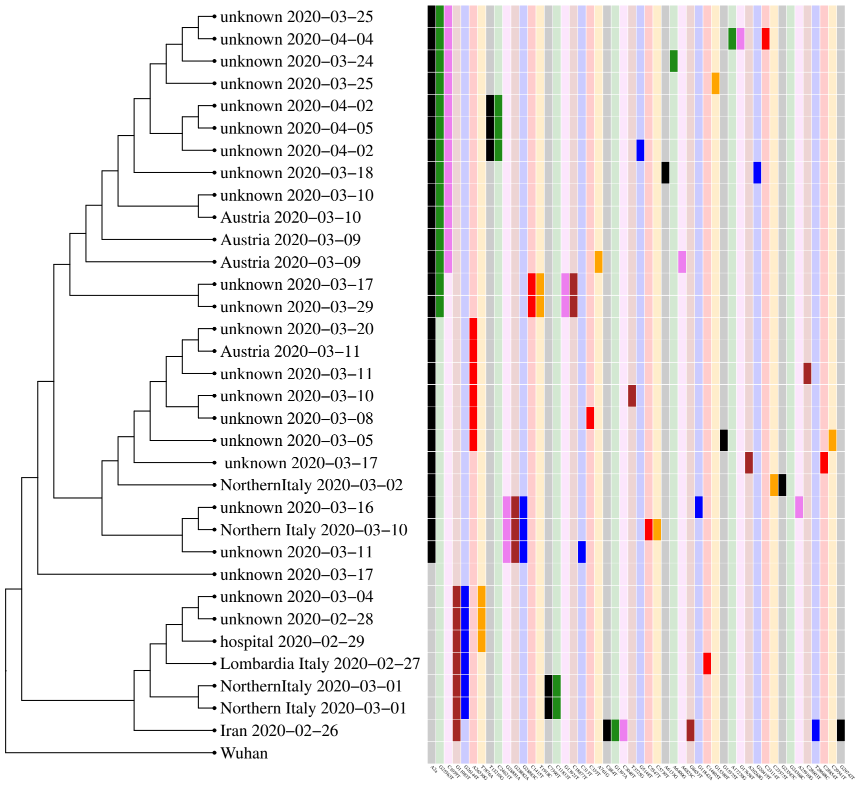

FIG S3. **Phylogenetic tree with Norwegian sequences.** The tree shows the Norwegian sequences with travel history metadata and mutations with respect to the Wuhan standard reference. Notice the match between the haplotype A2a2a and travel history in Austria.
