# Supplementary material for: Correction: SARS-CoV-2 transmission routes from genetic data: A Danish case study
Source: PLoS One. 2021 Dec 21;16(12):e0261892. doi: 10.1371/journal.pone.0261892 (PMC8691616; doi:10.1371/journal.pone.0261892)
Supplement: S2 File — (PDF) [file pone.0261892.s002.pdf]

PLOS ONE

RESEARCH ARTICLE

SARS-CoV-2 transmission routes from genetic data: A Danish case study

Andreas Bluhm[☯], **Matthias Christandl**[☯]*, **Fulvio Gesmundo**[☯], **Frederik Ravn Klausen**[☯], **Laura Mančinska**[☯], **Vincent Steffan**[☯], **Daniel Stilck França**[☯], **Albert H. Werner**[☯]

Department of Mathematical Sciences, University of Copenhagen, Copenhagen, Denmark

☯ These authors contributed equally to this work.
* christandl@math.ku.dk

Check for updates

OPEN ACCESS

Citation: Bluhm A, Christandl M, Gesmundo F, Ravn Klausen F, Mančinska L, Steffan V, et al. (2020) SARS-CoV-2 transmission routes from genetic data: A Danish case study. PLoS ONE 15(10): e0241405. https://doi.org/10.1371/journal.pone.0241405

Editor: Peter Gyarmati, University of Illinois College of Medicine, UNITED STATES

Received: June 26, 2020

Accepted: October 14, 2020

Published: October 29, 2020

Copyright: © 2020 Bluhm et al. This is an open access article distributed under the terms of the Creative Commons Attribution License, which permits unrestricted use, distribution, and reproduction in any medium, provided the original author and source are credited.

Data Availability Statement: All relevant data about the sequences used can be found within the manuscript and its Supporting Information files. The sequences themselves are available from https://www.gisaid.org/. The R code used is available from https://github.com/qmath/phylo_qmath.

Funding: All authors acknowledge financial support from VILLUM FONDEN via the QMATH Centre of Excellence (Grant no. 10059). AHW is supported by VILLUM FONDEN with a Villum Young

Abstract

Background

The first cases of COVID-19 caused by the SARS-CoV-2 virus were reported in China in December 2019. The disease has since spread globally. Many countries have instated measures to slow the spread of the virus. Information about the spread of the virus in a country can inform the gradual reopening of a country and help to avoid a second wave of infections. Our study focuses on Denmark, which is opening up when this study is performed (end-May 2020) after a lockdown in mid-March.

Methods

We perform a phylogenetic analysis of 742 publicly available Danish SARS-CoV-2 genome sequences and put them into context using sequences from other countries.

Results

Our findings are consistent with several introductions of the virus to Denmark from independent sources. We identify several chains of mutations that occurred in Denmark. In at least one case we find evidence that the virus spread from Denmark to other countries. A number of the mutations found in Denmark are non-synonymous, and in general there is a considerable variety of strains. The proportions of the most common haplotypes remain stable after lockdown.

Conclusion

Employing phylogenetic methods on Danish genome sequences of SARS-CoV-2, we exemplify how genetic data can be used to trace the introduction of a virus to a country. This provides alternative means for verifying existing assumptions. For example, our analysis supports the hypothesis that the virus was brought to Denmark by skiers returning from Ischgl. On the other hand, we identify transmission routes which suggest that Denmark was part of a network of countries among which the virus was being transmitted. This challenges the common narrative that Denmark only got infected from abroad. Our analysis concerning

Investigator Grant (Grant No. 25452). See https://veluxfoundations.dk/en for the webpage of VILLUM FONDEN. The authors received no specific funding for this work. The funders had no role in study design, data collection and analysis, decision to publish, or preparation of the manuscript.

Competing interests: The authors have declared that no competing interests exist.

the ratio of haplotypes does not indicate that the major haplotypes appearing in Denmark have a different degree of virality.

Introduction

According to peer-reviewed studies, the first cases of COVID-19 were reported in the city of Wuhan (China) at the first of December 2019 and a new virus, named SARS-CoV-2, was later identified as its origin [1]. At the time of writing, the pandemic is ongoing and has spread to more than 180 countries [2].

The first European case was reported in France on January 24, 2020 [3]. Italy confirmed its first two cases only a few days later on January 31 [4]. Austria reported its first cases on February 25 [5]. In March, Europe was the center of the global pandemic with many European countries introducing lockdown measures and travel restrictions. Early on, the ski area of Ischgl in Tyrol, Austria, was identified as a transmission hot-spot by some countries, so Iceland already declared it a risk area on March 5 [6]. Quarantine measures in Ischgl, however, were only imposed on March 13 [7].

Denmark confirmed its first case on February 27 after a man who returned home on February 24 from skiing holidays in Northern Italy had tested positive [8]. The second case was confirmed on February 28 and it was also associated to a traveller returning home from Northern Italy [9]. The number of cases kept increasing, and there was increased suspicion of community transmission after two cases had been confirmed at a local high school on March 8 [10].

During this first phase of the pandemic Denmark had issued travel warnings for certain high-risk areas. On March 2, Denmark advised against all travel to Northern Italy [11]. On March 10, Denmark additionally advised against travel to the Austrian state of Tyrol, as many travellers had tested positive after returning home from ski holidays in Ischgl. [12].

On March 11, the Danish prime minister Mette Frederiksen announced a lockdown, which happened in several stages and included closures of borders and schools [13]. Overall, the measures were not as severe as in some other European countries. On April 6, the prime minister announced that the first phase of reopening would start from April 14 [14]. The country has opened up further since.

As of May 26, there were 11,428 confirmed infections and 563 deaths in Denmark in connection with the disease [15]. From March 12, only people with serious symptoms and people in risk groups were tested. Since April 1, the number of tests has been increased [16].

In this work, we study all the publicly available genome sequences of the SARS-CoV-2 virus from Denmark as of May 26. The amount of sequences available makes Denmark a natural choice for a case study. Moreover, Denmark can serve as a prototype for a country which was internationally well-connected at the beginning of the pandemic and subsequently went into a strict lockdown. An investigation of the transmission routes of the virus in Denmark could therefore be used to understand the development in other countries as well.

For this investigation, we compare the Danish sequences used for our work to genome sequences from abroad. See S2 File for a list of sequences and their labs of origin. We use the mutations in the genomic data to identify transmission routes. These appear in the genetic data as sequences of consecutive mutations and can be thought of as a coarse-grained version of transmission chains where one cannot resolve the transmission between individuals, because some of the sequences might be identical. Our focus is on chains of mutations

highlighting the introduction of the virus to Denmark, its transmission within Denmark, and its spread to other countries.

Materials and methods

In this work, we use publicly available sequenced genome of the SARS-CoV-2 virus. In the following, we describe how we obtained and analyzed these sequences. For a flow chart of this process, see Fig 1.

Acquisition of samples

The sequences were downloaded from the GISAID EpiCoV database [17, 18] on May 26, 2020, including 742 Danish sequences. See the S2 File for a full list of sequences including their origins. From the available sequences, we selected those which we deemed of high quality and used them for our analysis. Specifically, we only consider sequences with at least 29,000 nucleotides having at most 300 unidentified nucleotides (N's). This corresponds to the requirement of having at most 1% unidentified nucleotides, which is also imposed by GISAID for sequences designated as high coverage. Similar cut-off values are used, for example, in [19]. Moreover, we only consider sequences that originate from a human host. After these steps, we were left with 582 Danish sequences, which we focus on in our analysis. As such our analysis is based on significantly more data as compared to the more global, but less Denmark-specific analysis by Nextstrain. Moreover, the analysis is carried out in greater detail. Nextstrain lists 132 Danish sequences for the relevant date range (retrieved on: August 21 2020).

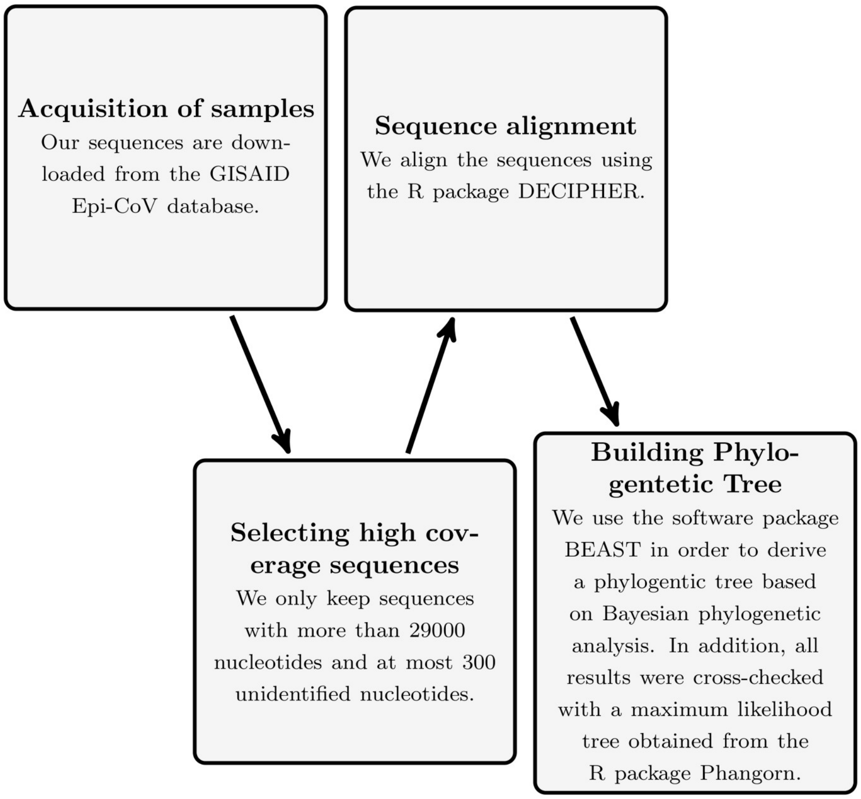

Fig 1. The tree building process as a flow chart.

https://doi.org/10.1371/journal.pone.0241405.g001

Tree inference

Based on the genetic data the phylogenetic trees of this manuscript where inferred from BEAST [20] in conjunction with the statistical software package R [21]. In particular, in a first step the sequences were aligned with the help of the R package DECIPHER [22, 23]. The resulting alignments were then further processed with BEAST in order to construct the phylogenetic trees. In all cases, the GTR + I + G model was used as the basis for the phylogenetic analysis. In order to cross-check this procedure, a maximum likelihood tree was built additionally, which in all cases supports the conclusions drawn from the BEAST results. The specific code we used can be found at https://github.com/qmath/phylo_qmath. We use the R-package *treedater* [24] to estimate mutation rates.

The mutations identified by this haplotype analysis were cross-validated via bootstrapping for the corresponding maximum-likelihood phylogenetic trees, with resulting bootstrap values consistent with the number of mutations defining the different clades. Moreover, we cross-checked our results with TreeTime [25], which led to similar tree-topologies. However, we do not require the additional time information provided by the TreeTime package in order to reach our conclusions.

Haplotypes and rooting conventions

We choose to root our trees with respect to the reference sequence NC-045512.2 (SARS-CoV-2 isolate Wuhan-Hu-1), which is also the reference for Nextstrain [26] and for [19, 27, 28]. This is unlikely to be the original sequence, as argued in [29]. The same work suggests rooting with respect to sequences found in bats and there is a debate in the literature concerning the most appropriate rooting strategy [29–31]. However, our haplotypes build upon the ones used in [27], which use this sequence as a reference. These haplotypes were in turn derived from (the original clades of) Nextstrain [26]. Furthermore, the sequences we consider were not collected before late February 2020. Therefore, the sequence NC-045512.2 from December 31, 2019 is sufficiently distant to serve as an outgroup to root our tree. In light of that, we believe that rooting with respect to NC-045512.2 has the advantage of allowing for a more straightforward comparison of the results of this work with [27] without compromising the quality of the displayed trees.

In Table 1, we list the mutations corresponding to the names we will use, following [27]. In addition, we list their names in the more recent Nextstrain convention [32] and the clades from the pangolin system that they are included in [28]. Finally, we list the corresponding amino acid changes and in which genes they can be found. See [33] for an overview of the SARS-CoV-2 genome.

Table 1. Naming of different haplotypes.

[27]	mutations	new Nextstrain	pang.	amino acid change
A2	C241T, C3037T, A23403G	19A/C241T/C3037T/A23403G	B	D614G in S
A2a	A2 + C14408T	19A/C241T/C3037T.20A	B.1	A2 + P4715L in Orf1ab
A2a1	A2a + GGG28881AAC	19A/C241T/C3037T.20A.20B	B.1.1	A2a + R203K + G204R in N
A2a2	A2a + G25563T	19A/C241T/C3037T.20A/G25563T	B.1	A2a + Q57H in Orf3a
A2a2a	A2a2 + C1059T	19A/C241T/C3037T.20A.20C	B.1	A2a2 + T265I in Orf1a

List of relevant haplotypes with their definition in terms of mutations as well as their new Nextstrain label. We note that both the reference string used and all the haplotypes listed have the four mutations specified as haplotype A in [27]. We therefore do not list those. The second to last column is the label for the currently identified pangolin lineage clades they are included in. The last column gives the corresponding amino acid changes and the genes they occur in.

https://doi.org/10.1371/journal.pone.0241405.t001

To get an overview of mutations prevalent in Denmark, we identify positions where sufficiently many of the analyzed sequences exhibit a substitution or a deletion as compared to the reference sequence. For a better overview and readability we choose different thresholds depending on the context. We analyze the co-occurrence of the new mutations with previously identified haplotypes from [27] and with each other in the entire worldwide data set.

Results

In this section, we review our results. After a general overview of the mutations over time, we study three different types of mutations in more detail: First, we consider mutations which were present in some region of the world and appeared in Denmark at some point. Second, we look at chains of mutations which only appear in Denmark. Finally, we look at mutations for which a Danish origin is predominant. The mutations we identify here are used subsequently in the Discussion section to analyze the spread from, to and within Denmark.

Distribution of mutations over time in Denmark

Let us now investigate the ratio of the haplotypes over time. The most common haplotypes in Denmark are A2a2a and A2a1. Fig 2 shows in the top panel the relative weight of those haplotypes over time with a seven-day rolling average. In the lower panel we plot the seven-day rolling average of the number of sequences. We observe a larger fraction of A2a1, which is associated to Italy, before the lockdown. From the onset of the lockdown, the fractions stay rather stable in time with A2a2a, which we discuss in detail below, making up around 70%. A lower number of sequences may be interpreted as a larger error bar on the haplotype percentages, making the haplotype distribution in time consistent with constant proportions.

The mutation rate we infer from the Danish data is consistent with the $6 \cdot 10^{-4}$ nucleotides/genome/year found in [19]. Please see [19, Table 1] for an overview of mutation rates for SARS-CoV-2 obtained in the literature.

Mutations from other regions appearing in Denmark

In the following, we study haplotypes present in Denmark which are also common in other countries. The aim is to identify from where they have been introduced to Denmark. We start with the haplotypes most common in the Danish data and proceed with specific examples of mutations less prevalent in Denmark. For an overview we refer to S1 Fig in S1 File.

A2a2a: A common mutation in Denmark and Ischgl. Approximately 70% of the available Danish sequences have haplotype A2a2a. This makes it the most common haplotype in our Danish data with 405 out of 582 sequences. In our complete data set, we see that 4343 out of 20239 sequences have this haplotype (see S1 Table in S1 File), with sequences originating from the US, Denmark, the UK, Australia, France and other countries. This haplotype was already reported in [27] where the authors point out that travelers from Austria had the haplotype A2a2 together with the mutation C1059T which is the definition of A2a2a. The haplotype A2a2 corresponds to an amino acid change Q57H in Orf3a as compared to A2a and the haplotype A2a2a corresponds to an amino acid change T265I in Orf1a as compared to A2a2 (see also Table 1). Both mutations have already been studied in [34].

In the following we first give evidence that some of the sequences with A2a2a originate from the skiing area of Ischgl in the region of Tyrol, Austria, by identifying specific chains of mutations. We will then argue that most, but likely not all of this haplotype comes from that area.

In order to identify a specific chain of mutations, we will look at mutations that occur in addition to A2a2a. Consider therefore mutation A6825C which corresponds to the amino acid

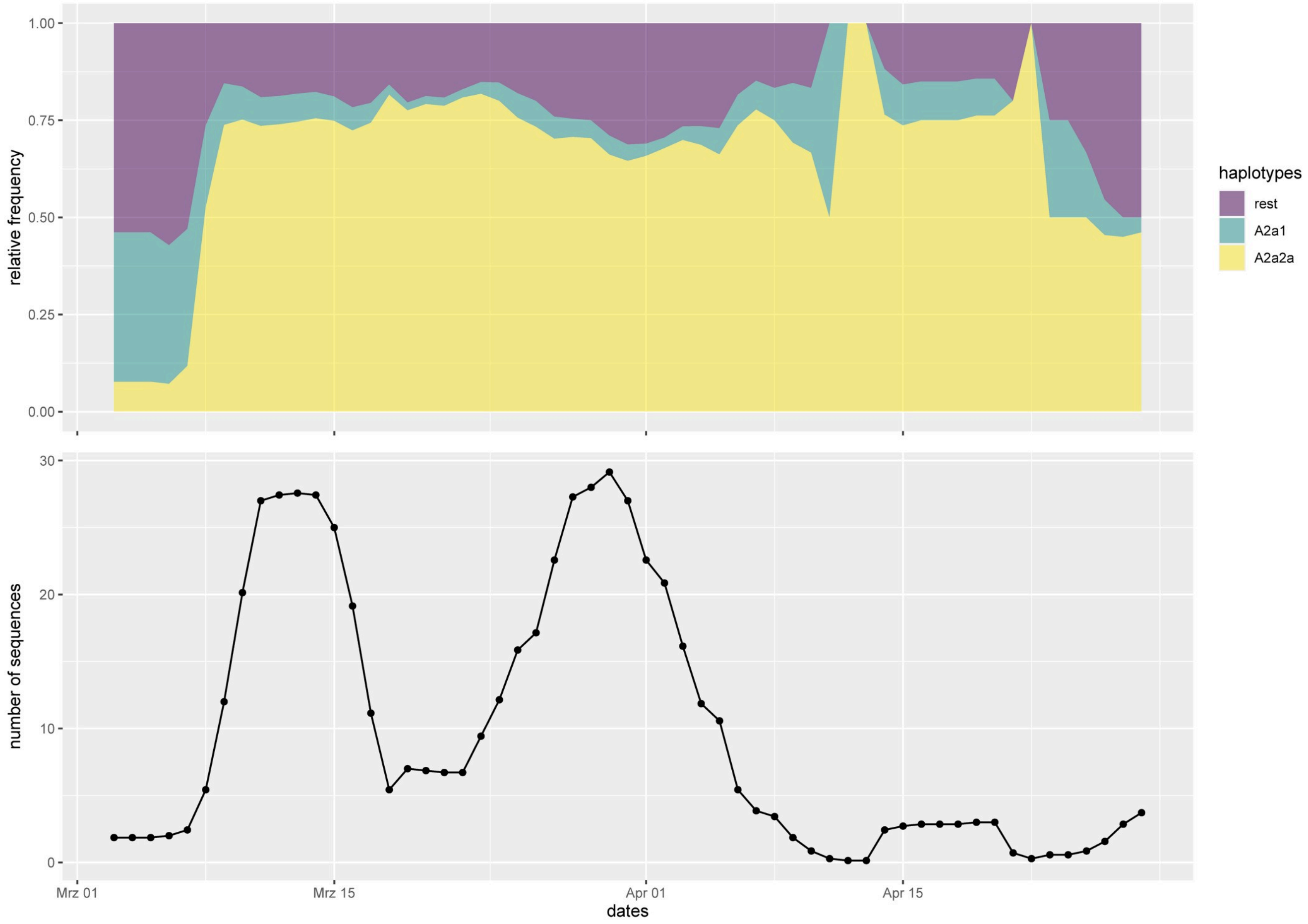

Fig 2. Haplotypes over time. Top panel: relative frequencies of major haplotypes in Denmark over time; bottom panel: total number of sequences over time. Both graphs are based on seven-day rolling averages.

https://doi.org/10.1371/journal.pone.0241405.g002

change N2187T in Orf1a and which is seen in nine sequences that have A2a2a worldwide. These include six Danish ones, while the others are from Austria, Norway and Scotland. The Norwegian sequence can be traced with metadata to Austria. The Norwegian sequence is dated to March 9, which makes it likely that this mutation has been present in Austria prior to that date. It is therefore consistent with the hypothesis that the Danish sequences, the first of which also is dated to March 9, originate from Austria. Since the Austrian sequence is from Ischgl, a spread from Ischgl, a tourist skiing destination, seems likely.

Similarly, the mutation G15380T (corresponding to S5039L in Orf1a) appears with haplotype A2a2a in 32 sequences worldwide. Among those 32 are 16 of Danish origin. Of the remaining ones with A2a2a, there are eight Austrian sequences. All of them stem from the region of Tyrol and in particular six are from Ischgl.

In order to argue that most Danish sequences with A2a2a originate from Austria, we first observe that the ratio of sequences with A2a2 versus A2a2a is close to 1 in Germany, Denmark, Norway, Austria, Iceland, Sweden and Switzerland what regards European countries, and lower in other European countries such as the UK, France and the Netherlands from which significant travel to Denmark would be expected. Since the number of Danish sequences with A2a2a is high even when restricting to the time around the onset of the lockdown, there must

have been multiple introductions of A2a2a to Denmark, and it therefore seems unlikely that this could have happened from a country with a much different ratio than that of Denmark.

Tourism from European countries to Alpine ski resorts around February and March would provide a natural travel route for the virus, in particular to Denmark, Sweden, Norway and Germany. For Iceland, this assumption is supported by the travel information collected in [27].

The sequences from Switzerland have no travel histories, but location data shows that the Swiss sequences with A2a2a are mainly spread across the German-speaking part and that they are in particular not concentrated at one location. This makes it unlikely that there was a hotspot in a Swiss ski resort.

In contrast, the Austrian sequences can be mainly attributed to the skiing region of Ischgl. We refer to the Austrian data presented in S2 Fig of S1 File, where one sees that the haplotype A2a2a is mostly present in sequences from the ski village of Ischgl in the region of Tyrol, Austria, and the adjacent region of Vorarlberg.

Accordingly, it seems very plausible that Ischgl was indeed a hotspot for the transmission of the haplotype A2a2a to the aforementioned countries.

The Norwegian sequences have travel metadata and give further supporting evidence for this infection route. Here, three out of twelve sequences with the haplotype A2a2a also have recent travel history to Austria (the others having unknown travel history). This is shown in S3 Fig in S1 File. The Icelandic study [27] also associated the haplotype A2a2a with travel to Austria.

However, due to the abundance of the haplotype A2a2a in the world, it is likely that some portion of the sequences with the haplotype A2a2a are not part of transmission route through Ischgl. As an example we discuss A2a2a + G24368T in Subsection S1.4 of S1 File, which we identify as likely originating from the UK.

A2a1: A common mutation in Denmark and Italy. While the previous haplotype could be linked to Austria, we now proceed with a haplotype that can be traced back to a different country. The haplotype A2a1, which we consider now, appears 38 times in Denmark. Moreover, 36 sequences with haplotype A2a1 were found in the early targeted testing group (January 31-March 15) in the Icelandic study [27 Table 2]. Out of these, 29 had a travel history from Italy and three from Austria. Furthermore, the earliest Danish sequence (dated February 26) is from when there was only one confirmed case in Denmark. As reported in the news, this case has travel history to an Italian ski-area. It also has haplotype A2a1.

The triple deletion ATGA1605A with coincident mutation T514C. Both in the Danish and the worldwide data sets we observe sequences with a triple deletion at sites 1606–1608 (ATGA1605A) which is sometimes coincident with a substitution T514C (identified as A6 in [27 Table S3]). The triple deletion corresponds to the triple deletion ATGA1604A identified as haplotype A9 in [27 Table S3]. Note that [27 Table S3] places it at position 1604 rather than 1605, which seems to be a typo. The CoV-GLUE database confirms the deletion at the nucleotides where we find it [35].

Most of the sequences in our data set with the triple deletion ATGA1605A but without the substitution T514C are from the UK (293 out of 346). Noticeably, there are six sequences from early February (the remaining dated from earliest March 1). Five of these are from the UK and one is from France. In contrast, most of the sequences with both the deletion ATGA1605A and the substitution T514C are from the Netherlands (98 out of 138). In addition, the earliest of the sequences with both ATGA1605A and T514C are from the Netherlands as well. Therefore, we conclude it to be likely that the triple deletion originated in the UK and then spread to the Netherlands, where it picked up the mutation T514C. Interestingly, some of the UK sequences also exhibit mutation T514C. We deem that they originate from the UK thus

highlighting the multidirectional spread of the virus. In Denmark, we observe nine sequences with ATGA1605A, two of which additionally have T514C. These latter two Danish sequences are likely of Dutch origin. The mutation T514C is not shown in S1 Fig of S1 File, since it appears only twice in the Danish data. However, the sequences in question are those at the very bottom, with numbers EPI_ISL_444828|2020-03-11 and EPI_ISL_429295|2020-03-13.

Chains of mutations starting in Denmark

Now, we turn to chains of mutations which occurred inside Denmark. From S1 Fig in S1 File, one identifies several such chains of mutations. Here we report two of the most pronounced.

Chain of mutations starting at C15842A. The first chain we consider starts at C15842A. The corresponding phylogenetic tree with an overview of the associated haplotypes for this mutation can be found in Fig 3. There are 20 sequences with the mutation C15842A and the haplotype A2a2a worldwide and they are all of Danish origin.

From the 20 (all Danish) sequences with A2a2a and C15842A, there are 17 which also have the mutation C12781T. Furthermore, of the sequences that have both the mutations C15842A (T5193N in Orf1a) and C12781T (synonymous), there are eight which in addition have the non-synonymous mutation G22103C (G181R in the spike protein). Another four sequences have the mutation A23975G instead and finally, there are two which have C25499T. Some of the previously mentioned sequences have additional mutations. The longest chain of mutations appearing at least twice has length three (not counting the mutations composing the haplotype A2a2a; see Fig 3).

Chain of mutations starting at C1302T. The Danish sequences with haplotype A2a2a frequently show the mutation C1302T. It is non-synonymous and corresponds to amino acid change T346I in Orf1a. We will argue that this mutation originated in Denmark and that it mutated further in Denmark as well as spread to other countries. See Fig 4 for the phylogenetic tree corresponding to this mutation.

In order to see that this mutation spread further from Denmark, note that worldwide there are 115 sequences with the mutation C1302T co-occurrent with the haplotype A2a2a, 103 of which are Danish. The remaining ones are Latvian (1), Icelandic (5) and Swedish (6). The travel histories of the five Icelandic sequences show that two have traveled to Denmark (as first reported in [27]), while the other cases do not contain travel information. Of the six Swedish sequences, one is from Uppsala dated to March 12 while the five others are from Norrbotten (in the north of Sweden) dated from March 24 until April 2. The earliest Danish sequence with C1302T is from March 3. Whereas our analysis does not completely exclude that the virus spread from Sweden or Latvia to Denmark, we believe that the earlier date of the Danish sequence, together with the high abundance in Denmark, makes it very likely that it originated in Denmark and further spread from Denmark. See also Fig 5(a) for an illustration of the international presence of the mutation.

In order to see that the strain A2a2a + C1302T further mutated in Denmark we inspect the corresponding clade of the Danish tree in S1 Fig of S1 File (see also Fig 4). Ten of the sequences have C11074T (a combination which is not found outside of Denmark. The eleventh sequence in this clade has an N at 11074.). Of these, six have the mutation C29095T. Three of them moreover have the mutation A9280G, whereas two have the mutation C619T (this is only visible in Fig 4 due to a threshold of three when displaying mutations in S1 Fig of S1 File). Of the ones with mutation A9280G, two have a mutation at C7164T. Some of the sequences have additional single mutations. We have thus identified the Danish chains of mutations in Fig 5(b).

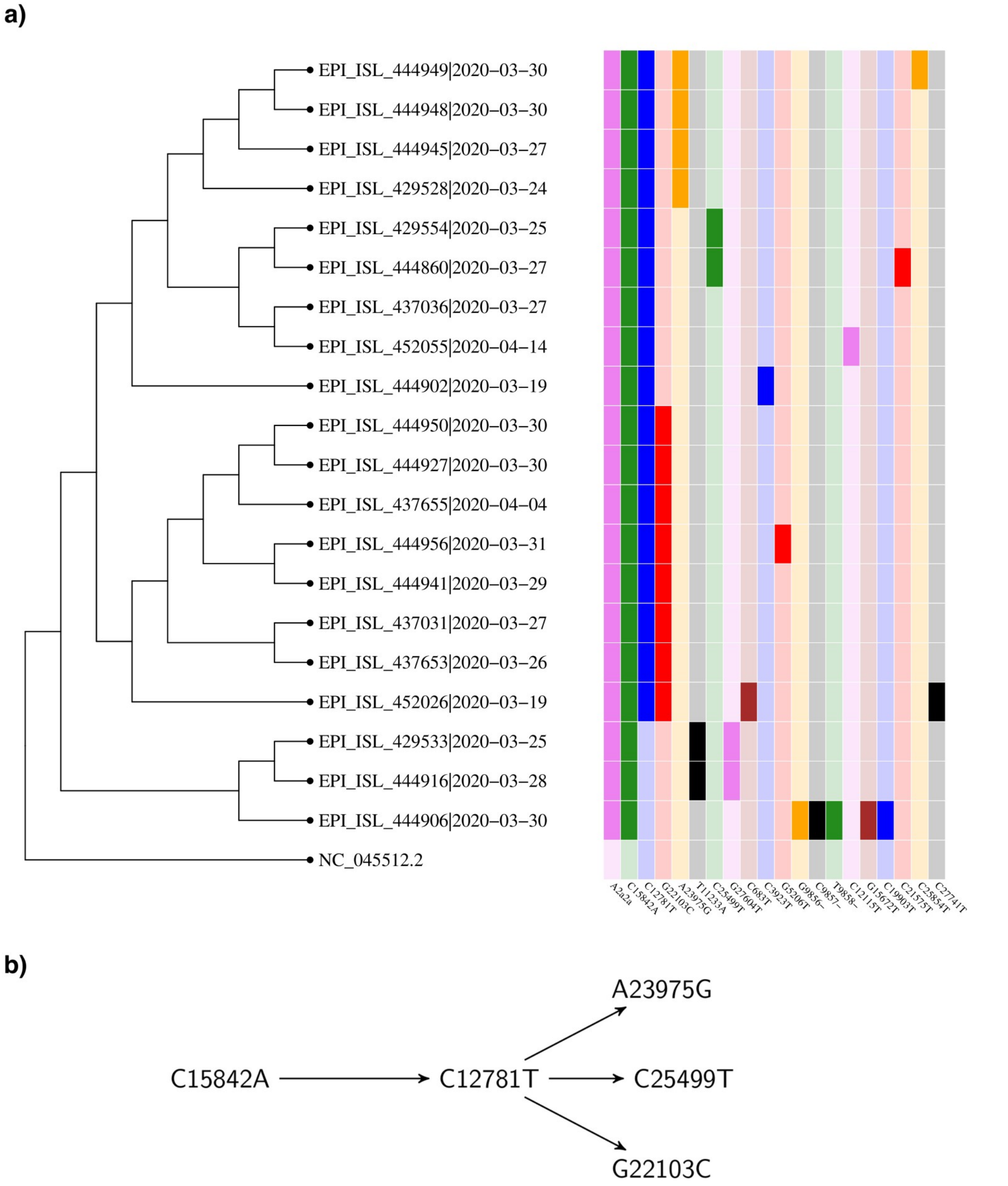

Fig 3. (a) Phylogenetic tree for sequences containing mutation C15842A. From the representation one can read of the chain mutations starting at C15842A. The second mutation shown is C15842A, followed by C12781T. After that, it trifurcates into G22103C, A23975G and C25499T. (b) Chain of mutations starting at C15842A.

https://doi.org/10.1371/journal.pone.0241405.g003

Discussion

We will start the discussion with the ratio of haplotypes over time before considering the different types of transmission routes we have found. We will conclude the section with an outlook.

Haplotypes over time

During the initial period of the introduction of the virus to Denmark from different sources the percentages of the different major haplotypes change: From a larger proportion of haplotype A2a1 associated to Italy to a 70% proportion A2a2a associated to Ischgl. After lockdown,

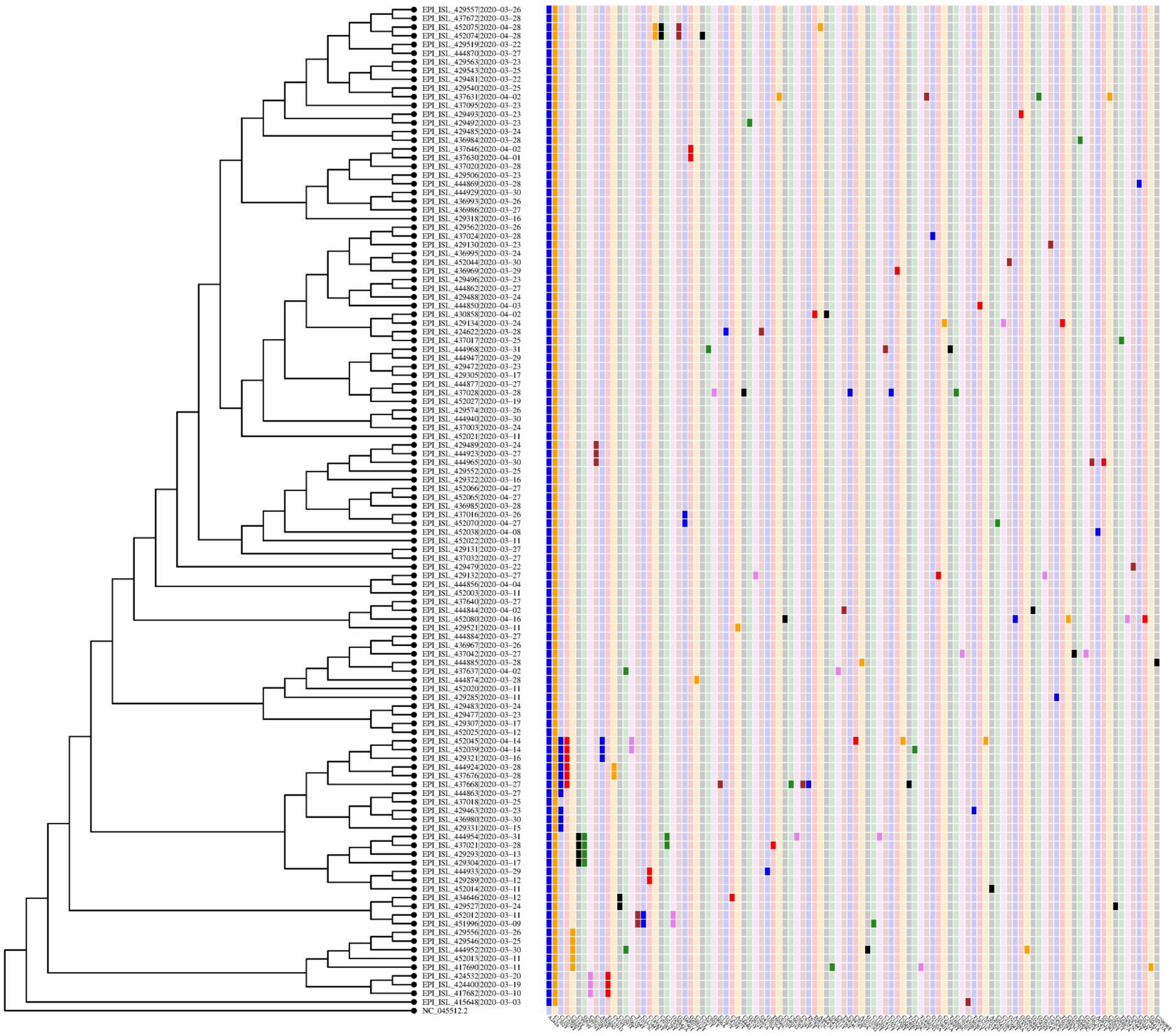

Fig 4. Phylogenetic tree for sequences containing mutation C1302T. From the representation one can read of the chain mutations starting at C1302T. The second mutation is C11074T, followed by C29095T. Subsequently, the chain bifurcates into C619T and A9280G followed by C7164T.

https://doi.org/10.1371/journal.pone.0241405.g004

however, we do not observe a significant change in the proportions anymore. Therefore, we find no evidence for different virality. We find no clear pattern in individual mutations of the strains appearing in Denmark either. Therefore, we suspect that they are consistent with random mutation events.

After the research on this study had been concluded, a possible difference in virality of the occurrence/non-occurrence of mutation D614G in the spike protein has been discussed in [36, 37]. We point out that nearly all studied Danish sequences have this mutation (see S1 Table in S1 File).

Introduction to Denmark

We now discuss the question of how the virus came to Denmark. Our phylogenetic analysis shows that around 70% of the Danish sequences have haplotype A2a2a. By comparing the

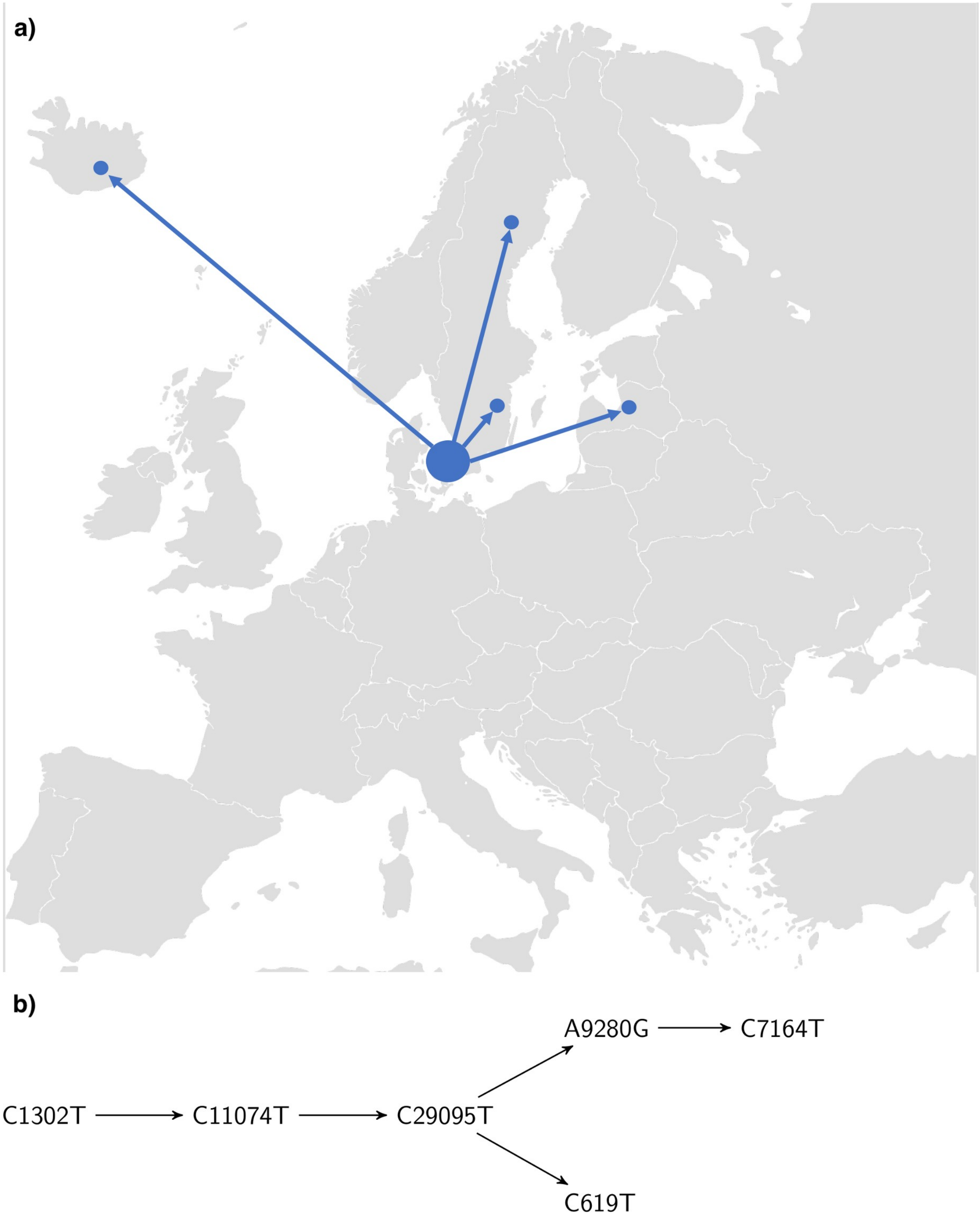

Fig 5. (a) Spread of the strain with haplotype C1302T. The figure shows the likely spread of the strain with haplotype C1302T from Denmark to other Northern European countries. Within Denmark, it also mutated further and gave rise to the chain of mutations displayed in (b). (b) Chain of mutations starting at C1302T.

https://doi.org/10.1371/journal.pone.0241405.g005

distribution of haplotypes across different countries, by utilizing date information, and, for some international sequences, also location data as well as travel histories, we conclude that the majority of the Danish sequences with A2a2a originate directly or indirectly from Ischgl. This is illustrated with examples of specific chains of mutations from Ischgl. Our observation that a large proportion of Danish sequences originate in Ischgl is not unexpected given the public knowledge of travel histories [38]. Our analysis, however, can be regarded as an independent cross-check of this existing narrative.

The remaining portion of the sequences is consistent with multiple entries from other countries, among them Italy, the UK and the Netherlands. We have illustrated this with example chains of mutations which can be associated to those countries. In the case of Italy, this is based on the haplotype A2a1, in the case of the UK, it is a specific mutation on top of haplotype A2a2a and in the case of the Netherlands, it is a mutation in addition to a well-known triple deletion. These conclusions are consistent with the testing results in mid-March [39].

From this point of view, our genomic analysis cross-validates the public statements and supports findings in the Iceland study [27] that indicate that Ischgl was a hotspot earlier than widely recognized.

Transmission routes inside Denmark

After its introduction to Denmark, the virus continued to mutate within the country. We have listed all mutations that appear at least three times inside Denmark in S2 Table in S1 File and also plotted them in S1 Fig of S1 File. We note that many more Danish chains of mutations can be identified from S1 Fig of S1 File.

In the results, we discussed two particularly pronounced chains of mutations based on this plot and Fig 3. For the two chains described in the results we conclude that they are chains of mutations that happened inside Denmark. We have chosen these two since these mutation chains only co-occur with the haplotype A2a2a in Denmark (except of the first mutation C1302T). This shows clearly how one can track the virus mutating as it spreads inside Denmark. The longest chain that we conclude happened inside Denmark is five mutations long and consists of the mutations [C1302T → C11074T → C29095T → A9280G → C7164T]. These mutations took place in a period from before March 15 to before April 14 based on the dating of the sequences. The average mutation rate we obtain is consistent within error bars with the $6 \cdot 10^{-4}$ nucleotides/genome/year obtained in [19].

Transmission out of Denmark

Not only did the virus follow the travel routes into Denmark, it also spread from Denmark. For the mutation C1302T, based on its high prevalence in Denmark compared to the rest of the world together with the travel histories of the Icelandic cases, we conclude that it appeared first in Denmark and spread from there to Sweden, Latvia and to Iceland. Some reservations remain since the Swedish data in GISAID is very limited with only 163 sequences as of May 26. Further, the chain of mutations described shows how the virus has spread extensively within Denmark and mutated at least four times after that.

Hence we see indications that the virus has mutated several times inside Denmark and spread from Denmark, as illustrated in Fig 5. We have listed and discussed the most common mutations. As we show in S1 File, some of the mutations we see seem to have occurred independently elsewhere, in particular in the UK, which has a large number of sequences in GISAID. An example of this is the mutation C7011T.

Outlook

The conclusions above are based on a rather large number of high quality Danish sequences with date information as well as on sequences from other countries some of which have more metadata. Even though we do not have firm knowledge of the representativity of the Danish sequences, we can assert that they cover the entire time from the first identified case up to May 9. In order to confirm the analysis of the proportion of haplotypes seen, it would be important to supplement this with information about the representativity of the analysed data set or obtain a more representative sample. At the same time, our analysis also shows how to effectively incorporate metadata (date, country of origin or travel history) in such an analysis.

We have used the SARS-CoV-2 genomic data to identify transmission routes, thus highlighting the potential of such methods for understanding the spread of the virus in a population. Although here we present a case study for Denmark, a similar analysis could be carried out for outbreaks in other countries, regions or even smaller units such as hospitals. If sufficient data is available, such methods can also be used to identify transmission chains between individuals as done e.g. in [27]. If the genomic data is available in real-time, such an analysis can inform mitigation measures even during an ongoing outbreak, for instance supplementing traditional methods such as contact tracing.

Supporting information

S1 File. Discussion of additional Danish mutations and supplementary tables and phylogenetic trees.
(PDF)

S2 File. GISAID acknowledgements. List of sequences from GISAID used and their submitting laboratories.
(PDF)

Acknowledgments

We thank Judith Gottwein, Anders Krogh, Jakob Sture Madsen and Carsten Wiuf for valuable comments. We would like to thank everyone submitting sequenced genome data to GISAID, in particular Statens Serum Institut, with whom we shared an earlier version of this manuscript, and Mads Albertsen's lab. A full list of the contributors can be found in S2 File.

Note

During compilation of this work, we became aware of concurrent work, which was announced here: TV Avisen, DR. Ny genforskning afsører: Sädan endte Midt-og Vestjylland med at blive hotspot for corona; 2020 May 28 9pm. Available from: https://www.dr.dk/nyheder/indland/ny-genforskning-afsloerer-saadan-endte-midt-og-vestjylland-med-blive-hotspot-corona#! [cited 2020 May 29].

Author Contributions

Conceptualization: Andreas Bluhm, Matthias Christandl, Frederik Ravn Klausen, Laura Mančinska, Daniel Stilck França, Albert H. Werner.

Data curation: Fulvio Gesmundo.

Investigation: Frederik Ravn Klausen, Laura Mančinska.

Methodology: Andreas Bluhm, Daniel Stilck França, Albert H. Werner.

Software: Fulvio Gesmundo, Vincent Steffan.

Supervision: Matthias Christandl.

Visualization: Vincent Steffan.

Writing – original draft: Andreas Bluhm, Matthias Christandl, Fulvio Gesmundo, Frederik Ravn Klausen, Laura Mančinska, Vincent Steffan, Daniel Stilck França, Albert H. Werner.

References

1. Huang C, Wang Y, Li X, Ren L, Zhao J, Hu Y, et al. Clinical features of patients infected with 2019 novel coronavirus in Wuhan, China. Lancet. 2020; 395(10223):497–506. https://doi.org/10.1016/S0140-6736(20)30183-5 PMID: 31986264
2. WHO. Coronavirus disease (COVID-19) Situation Report—116. Geneva, Switzerland: World Health Organization; 2020. Available from: https://www.who.int/docs/default-source/coronaviruse/situation-reports/20200515-covid-19-sitrep-116.pdf?sfvrsn=8dd60956_2.
3. Bernard Stoecklin S, Rolland P, Silue Y, Mailles A, Campese C, Simondon A, et al. First cases of coronavirus disease 2019 (COVID-19) in France: surveillance, investigations and control measures, January 2020. Euro Surveill. 2020; 25(6):2000094. https://doi.org/10.2807/1560-7917.ES.2020.25.6.2000094 PMID: 32070465
4. WHO. Coronavirus disease (COVID-19) Situation Report—11. Geneva, Switzerland: World Health Organization; 2020. Available from: https://www.who.int/docs/default-source/coronaviruse/situation-reports/20200131-sitrep-11-ncov.pdf?sfvrsn=de7c0f7_4.
5. WHO. Coronavirus disease (COVID-19) Situation Report—37. Geneva, Switzerland: World Health Organization; 2020. Available from: https://www.who.int/docs/default-source/coronaviruse/situation-reports/20200226-sitrep-37-covid-19.pdf?sfvrsn=2146841e_2.
6. Embætti landlæknis. Skíðasvæðið Ischgl í Austurríki í hóp skilgreindra áhættusvæða; 2020 Mar 5. Available from: https://www.landlaeknir.is/um-embaettid/frettir/frett/item39457/skidasvaedid-ischgl-i-austurriki-i-hop-skilgreindra-ahaettusvaeda [cited 2020 May 28].
7. Amt der Tiroler Landesregierung. Ischgl-Chronologie; 2020 May 5. Available from: https://www.tirol.gv.at/meldungen/meldung/artikel/ischgl-chronologie/ [cited 2020 May 28].
8. TV2. Dansker smittet med coronavirus; 2020 Feb 27. Available from: https://nyheder.tv2.dk/samfund/2020-02-27-dansker-smittet-med-coronavirus [cited 2020 May 15].
9. Sundhedsstyrelsen nyheder. En person, der er blevet undersøgt på Rigshospitalet, er det andet bekræftede tilfælde af COVID-19 i Danmark; 2020 Feb 28. Available from: https://www.sst.dk/da/Nyheder/2020/En-person-der-er-blevet-undersoegt-paa-Rigshospitalet-er-det-andet-bekraeftede-tilfaelde-af-COVID-19 [cited 2020 May 15].
10. Danish Broadcasting Corporation. DR news—Gymnasium lukker efter nyt tilfælde af corona-smitte; 2020 Mar 8. Available from: https://www.dr.dk/nyheder/indland/gymnasium-lukker-efter-nyt-tilfaelde-af-corona-smitte [cited 2020 May 15].
11. Sundhedsstyrelsen nyheder. COVID-19: Nye anbefalinger til borgere; 2020 Mar 3. Available from: https://www.sst.dk/da/nyheder/2020/nye-anbefalinger-fra-sundhedsstyrelsen-om-at-blive-hjemme-i-to-uger [cited 2020 May 15].
12. Statsministeriet Pressemøde. Pressemøde om COVID-19 den 10 Marts 2020; 2020 Mar 10. Available from: http://www.stm.dk/_p_14919.html [cited 2020 May 15].
13. Statsministeriet Pressemøde. Pressemøde om COVID-19 den 11 Marts 2020; 2020 Mar 11. Available from: http://www.stm.dk/_p_14920.html [cited 2020 May 15].
14. Statsministeriet Pressemøde. Pressemøde om COVID-19 den 6 April 2020; 2020 Apr 6. Available from: http://www.stm.dk/_p_14938.html [cited 2020 May 15].
15. Statens Serum Institut. Overvågningsdata; 2020 May 26. Available from: https://files.ssi.dk/Data-Epidemiologiske-Rapport-26052020-snn6 [cited 2020 June 10].
16. SSI. COVID-19 i Danmark—Epidemiologisk overvågningsrapport. Statens Serum Institut; 2020 May 12. Available from: https://files.ssi.dk/COVID19-overvaagningsrapport-12052020-2-8ft5.
17. Elbe S, Buckland-Merrett G. Data, disease and diplomacy: GISAID's innovative contribution to global health. Glob Chall. 2017; 1(1):33–46. https://doi.org/10.1002/gch2.1018
18. Shu Y, McCauley J. GISAID: Global initiative on sharing all influenza data—from vision to realty. Euro Surveill. 2017; 22(13):30494. https://doi.org/10.2807/1560-7917.ES.2017.22.13.30494

19. van Dorp L, Acman M, Richard D, Shaw LP, Ford CE, Ormond L, et al. Emergence of genomic diversity and recurrent mutations in SARS-CoV-2. Infect Genet Evol. 2020; p. 104351. https://doi.org/10.1016/j.meegid.2020.104351 PMID: 32387564

20. Bouckaert R, Vaughan TG, Barido-Sottani J, Duchêne S, Fourment M, Gavryushkina A, et al. BEAST 2.5: An advanced software platform for Bayesian evolutionary analysis. PLoS Comput Biol. 2019; 15 (4):e1006650. https://doi.org/10.1371/journal.pcbi.1006650 PMID: 30958812

21. R Core Team. R: A Language and Environment for Statistical Computing. Vienna, Austria; 2017. Available from: https://www.R-project.org/.

22. Wright ES. DECIPHER: harnessing local sequence context to improve protein multiple sequence alignment. BMC Bioinformatics. 2015; 16(322). https://doi.org/10.1186/s12859-015-0749-z PMID: 26445311

23. Wright ES. Using DECIPHER v2.0 to Analyze Big Biological Sequence Data in R. R J. 2016; 8(1):352–359. https://doi.org/10.32614/RJ-2016-025

24. Volz EM, Frost SDW. Scalable relaxed clock phylogenetic dating. Virus Evol. 2017; 3(2). https://doi.org/10.1093/ve/vex025

25. Sagulenko P, Puller V, Neher RA. TreeTime: Maximum-likelihood phylodynamic analysis. Virus Evolution. 2018; 4(1). https://doi.org/10.1093/ve/vex042 PMID: 29340210

26. Hadfield J, Megill C, Bell SM, Huddleston J, Potter B, Callender C, et al. Nextstrain: real-time tracking of pathogen evolution. Bioinformatics. 2018; 34(23):4121–4123. https://doi.org/10.1093/bioinformatics/bty407 PMID: 29790939

27. Gudbjartsson DF, Helgason A, Jonsson H, Magnusson OT, Melsted P, Norddahl GL, et al. Spread of SARS-CoV-2 in the Icelandic Population. N Engl J Med. 2020; 382:2302–2315. https://doi.org/10.1056/NEJMoa2006100 PMID: 32289214

28. Rambaut A, Holmes EC, Hill V, O'Toole Á, McCrone J, Ruis C, et al. A dynamic nomenclature proposal for SARS-CoV-2 to assist genomic epidemiology. bioRxiv. 2020. https://doi.org/10.1101/2020.04.17.046086 PMID: 32669681

29. Forster P, Forster L, Renfrew C, Forster M. Phylogenetic network analysis of SARS-CoV-2 genomes. Proc Natl Acad Sci U S A. 2020; 117(17):9241–9243. https://doi.org/10.1073/pnas.2004999117

30. Mavian C, Pond SK, Marini S, Magalis BR, Vandamme AM, Dellicour S, et al. Sampling bias and incorrect rooting make phylogenetic network tracing of SARS-COV-2 infections unreliable. Proc Natl Acad Sci U S A. 2020; 117(23):12522–12523. https://doi.org/10.1073/pnas.2007295117 PMID: 32381734

31. Forster P, Forster L, Renfrew C, Forster M. Reply to Sánchez-Pacheco et al., Chookajorn, and Mavian et al.: Explaining phylogenetic network analysis of SARS-CoV-2 genomes. Proc Natl Acad Sci U S A. 2020; 117(23):12524–12525. https://doi.org/10.1073/pnas.2007433117

32. Hodcroft E. Clade Naming & Definitions; 2020 June 2. Available from: https://github.com/nextstrain/ncov/blob/master/docs/clades.md [cited 2020 June 12].

33. Khailany RA, Safdar M, Ozaslan M. Genomic characterization of a novel SARS-CoV-2. Gene Rep. 2020; 19:100682. https://doi.org/10.1016/j.genrep.2020.100682

34. Issa E, Merhi G, Panossian B, Salloum T, Tokajian S. SARS-CoV-2 and ORF3a: Nonsynonymous Mutations, Functional Domains, and Viral Pathogenesis. mSystems. 2020; 5(3). https://doi.org/10.1128/mSystems.00266-20 PMID: 32371472

35. Singer J, Gifford R, Cotten M, Robertson D. CoV-GLUE: A Web Application for Tracking SARS-CoV-2 Genomic Variation; 2020. Available from: https://www.preprints.org/manuscript/202006.0225/v1.

36. Korber B, Fischer WM, Gnanakaran S, Yoon H, Theiler J, Abfalterer W, et al. Tracking Changes in SARS-CoV-2 Spike: Evidence that D614G Increases Infectivity of the COVID-19 Virus. Cell. 2020; 182 (4):812–827.e19. https://doi.org/10.1016/j.cell.2020.06.043. PMID: 32697968

37. Grubaugh ND, Hanage WP, Rasmussen AL. Making Sense of Mutation: What D614G Means for the COVID-19 Pandemic Remains Unclear. Cell. 2020; 182(4):794–795. https://doi.org/10.1016/j.cell.2020.06.040

38. SSI. COVID-19 i Danmark—Epidemiologisk overvågningsrapport. Statens Serum Institut; 2020 Mar 23. Available from: https://files.ssi.dk/COVID19-overvaagningsrapport-16032020.

39. SSI. COVID-19 i Danmark—Epidemiologisk overvågningsrapport. Statens Serum Institut; 2020 Mar 13. Available from: https://files.ssi.dk/COVID19-overvaagningsrapport-13032020.
